# First detection of highly pathogenic H5N6 avian influenza virus on the African continent

**DOI:** 10.1080/22221751.2020.1757999

**Published:** 2020-05-07

**Authors:** Ismaila Shittu, Alice Bianco, Dorcas Gado, Nicodemus Mkpuma, Lanre Sulaiman, Agnes Laleye, Federica Gobbo, Alessio Bortolami, Francesco Bonfante, Columba Vakuru, Clement Meseko, Alice Fusaro, David Shamaki, Olaniran Alabi, Calogero Terregino, Tony Joannis

**Affiliations:** a National Veterinary Research Institute, Vom, Nigeria; b Istituto Zooprofilattico Sperimentale delle Venezie, Padova, Italy; c Federal Ministry of Agriculture and Rural Development, Abuja, Nigeria

**Keywords:** Highly pathogenic avian influenza virus, H5N6, live bird market, Nigeria, duck

## Abstract

Since 2013, highly pathogenic avian influenza (HPAI) subtype H5N6 (clade 2.3.4.4) has been reported in wild birds and poultry in Asia as well as in other parts of the globe. In Africa, information on the presence of this virus subtype is lacking. This study reports the first detection of a HPAI (H5N6) virus (clade 2.3.4.4b) in a duck from a live bird market in Nigeria, whose genome is closely related to the European 2017–2018 H5N6 viruses, indricating a recent virus introduction into the African continent.

In January 2006, the first outbreak of a highly pathogenic avian influenza (HPAI) subtype H5N1 (clade 2.2) was detected in Nigeria [1]. Within two years from its introduction, the disease had spread to 67.6% of the states in the country in circumstances suspected to be linked to wild bird migration and/or trade [2]. The HPAI outbreaks resulted in colossal economic losses and public health concerns. Following the first epidemic wave of HPAI (2006–2007), in 2008, a distinct H5N1 virus (clade 2.2.1) was detected in ducks from live bird market (LBM) surveillance in Northeast (Gombe) Nigeria [2]. In January 2015, another HPAI H5N1 incursion (clade 2.3.2.1c) was reported from a LBM and from poultry farms in Lagos and Kano states, respectively [3]. A year later, in November 2016, a HPAI H5N8 virus of clade 2.3.4.4.b, responsible for one of the most devastating epizootic in poultry and wild birds in Europe [4], was detected in the northern state of Kano [5]. Since late-2017, a new reassortant HPAI clade 2.3.4.4b H5N6 has been reported in wild and domestic birds in Northern Europe. This study describes the first detection, in June 2019, of the same H5N6 subtype in a duck from LBM surveillance in Northwest (Sokoto) Nigeria (Figure 1).

**Figure 1.**
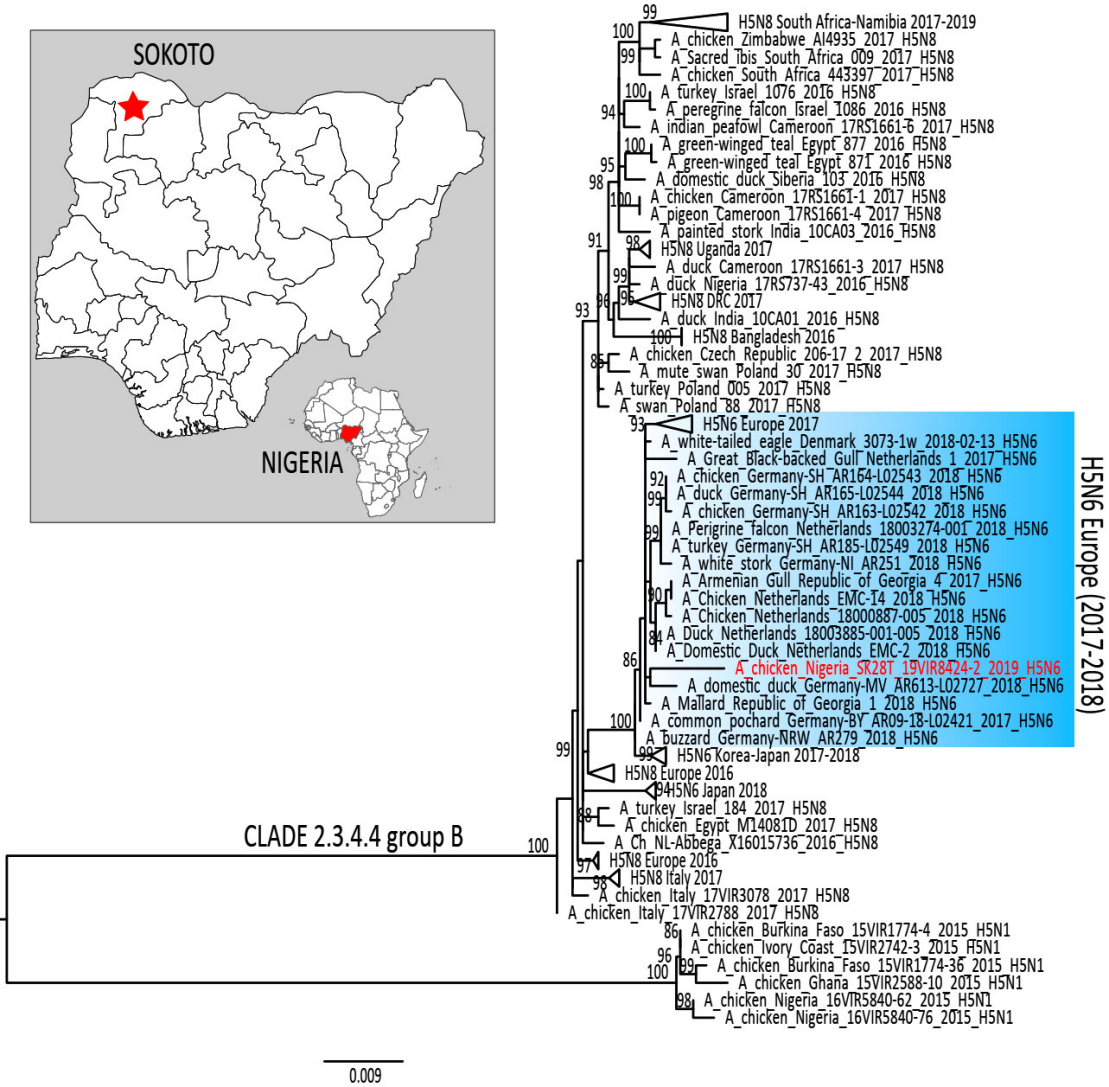
Maximum likelihood phylogenetic tree of the HA gene segment of A/duck/Nigeria/SK28T_19VIR8424-2/2019. H5N6 virus from Nigeria is marked in red. Blue rectangular highlights the European H5N6 cluster. The map shows the Nigerian state (red star), where the H5N6 was identified.

During a recent surveillance activity for HPAI conducted from late-June to mid-August 2019 by the Department of Veterinary and Pest Control Services of the Federal Ministry of Agriculture and Rural Development, Abuja, an isolate of avian influenza was identified and characterized. In the course of the surveillance activity, 3131 tracheal and cloacal samples collected from 13 bird species were tested at the NVRI using real-time RT–PCR for avian influenza virus (AIV) targeting the matrix (M) gene [6]. AIV M-gene positive samples were subjected to specific protocols for subtyping avian influenza viruses [7,8]. In addition, the M-gene positive samples were inoculated in 9–11-day-old embryonated chicken eggs of specific antibody-negative origin according to the standard procedure [9].

A H5N6 virus, designated A/duck/Nigeria/SK28T_19VIR8424-2/2019, was isolated and sent to the Istituto Zooprofilattico Sperimentale delle Venezie (IZSVe), Padua, Italy, for subtype confirmation. The intravenous pathogenicity index (IVPI) was conducted to assess the pathogenicity of the virus (supplementary data). To trace the virus origin and evaluate its genetic properties, whole-genome sequencing was performed on the isolate using an Illumina MiSeq platform (Illumina, San Diego, CA, USA) (supplementary data). The sequences were deposited at the GenBank under the accession numbers: MN889503-MN889510.

The maximum likelihood phylogenetic tree of the haemagglutinin (HA) gene segment obtained by using IQTREE (Figure 1) shows that the HPAI H5N6 virus detected in Nigeria in summer 2019 falls within genetic clade 2.3.4.4b [10] and groups together with European H5N6 viruses identified in wild and domestic birds in 2017–2018 (similarity range between 98.9% and 99%) (Supplementary Table 1). The phylogenetic trees of all other gene segments reflect the same topology as the HA phylogeny (data not shown).

The amino acid sequences of the haemagglutinin (HA) gene show that the H5N6 virus possesses a multi-basic cleavage site (PLREKRRKR*GLF) typical of HPAI viruses. Similarly, the IVPI test result, 2.89, confirmed the highly pathogenic nature of the isolate [9]. In the neuraminidase (NA) gene segment of the virus, mutation N403H removes a potential N-glycosylation site at position 403, at the level of the sialic acid-binding domain of the protein [11]. This mutation could potentially affect both the antigenic and the receptor binding properties of this strain. Nevertheless, haemagglutination inhibition assays conducted with ferret antisera generated against HPAI viruses belonging to the 2.3.4.4 clade, H5N6 A/Sichuan/26221/2014 (SICH-26221), H5N8 A/Fujian-Sanyuan/21099/2017XPR8 (FUJIAN-21099) and the recent 2.3.4.4b H5N8 A/turkey/Italy/17VIR576-11/2017 (ITALY-576) strains, recorded titers that were either identical to the ones observed against the homologous antigens or within 1 log_2_ difference. Genetic and antigenic data were generated and shared within the OFFLU network to contribute to the WHO biannual report of “Antigenic and genetic characteristics of zoonotic influenza viruses and development of candidate vaccine viruses for pandemic preparedness.”

Here, the first H5N6 clade 2.3.4.4.b introduction into Africa from Europe, most likely via wild birds is reported. HPAI H5N6 bearing the same gene constellation of the Nigerian strain, had already caused a total of 98 outbreaks in Europe between late-2017 and early 2019, of which 92% had occurred in wild birds [12]. This finding confirms our previous study, which recognized West Africa, and in particular Nigeria, as one of the most important hotspot for Gs/GD/96 HPAI H5Nx introduction into Africa [5]. Europe and West Africa are naturally connected by the Black Sea/Mediterranean flyway and, as suggested for previous virus spreads [5], wild migratory birds may have played an important role also in this new virus incursion. The virus may have circulated in the West African wild or domestic population for several months before its detection, highlighting the need to enhance surveillance, in particular in the areas close to wetlands. To prevent the spread of infection to other countries in the sub-region, coordinated control strategies have been applied, which include, but are not limited to, controlling the movement of poultry and poultry products with neighbouring countries. In addition, implementation of surveillance plans is mandatory to avoid possible gaps in monitoring the virus evolution and the epidemiological scenario.

Co-circulation of multiple subtypes (H5N8, H5N6 and H9N2) (T. Joannis pers. comm.) in the country may represent a diagnostic challenge, considering that new reassortant viruses could emerge, as recently reported in Egypt [13,14], and spread. Urgent actions to strengthen surveillance efforts, combined with eradication measures, adequate compensations and education of farmers are needed to contain and monitor virus spread and the emergence of novel viruses of animal and public health concern.

## Supplementary Material

Supplemental Material
