# Supplementary material for: First detection of highly pathogenic H5N6 avian influenza virus on the African continent
Source: Emerg Microbes Infect. 2020 May 7;9(1):886–8. doi: 10.1080/22221751.2020.1757999 (PMC7241522; doi:10.1080/22221751.2020.1757999)
Supplement: Supplemental Material [file TEMI_A_1757999_SM0701.docx]

**Materials and Methods**

**Classical and molecular tests**

Total RNA was extracted from the positive sample sent to IZSVe (i.e. the allantoic fluid obtained from virus isolation performed at NVRI) using the QIAamp Viral RNA Mini Kit (Qiagen, Hilden, Germany) and tested by Reverse Transcription (RT) PCR for Influenza A matrix gene [1], H5, H7 [2,3], H9 subtype [4] and neuraminidase subtyping [5].

Virus isolation was performed by inoculating 9-to-11-day-old embryonated specific pathogen free (SPF) hen’s eggs via the allantoic cavity.

**Genome Amplification and Sequencing**

We amplified the complete genome by using the SuperScript III One-Step RT-PCR System and Platinum Taq High Fidelity (Invitrogen, Carlsbad, CA, USA) using one pair of primers complementary to the conserved elements of the influenza A virus promoter as previously described [6] [7]. The sequencing library was prepared by using the Nextera DNA XT Sample preparation kit (Illumina, San Diego, CA, USA) and quantified by using the Qubit dsDNA High Sensitivity Kit (Invitrogen, Carlsbad, CA, USA). The High Sensitivity DNA Analysis Kit (Agilent Technologies, Alpharetta, GA, USA) was used to determine average fragment length. The indexed libraries were pooled in equimolar concentrations and sequenced in multiplex for 300bp paired-end on Illumina MiSeq, according to the manufacturer’s instructions.

**Illumina Sequencing Data Analysis**

FastQC version 0.11.2 (https://www.bioinformatics.babraham.ac.uk/projects/fastqc/) was used to assess read quality. Raw data were filtered by removal of reads with >10% of undetermined bases, reads with >100 bases with a Q score 80 bases were aligned against a reference genome by using BWA version 0.7.12 [8]. Picard-tools version 2.1.0 (http://picard.sourceforge.net) and GATK version 3.5 [9–11] were used to correct potential errors, realign reads around indels, and recalibrate base quality. LoFreq version 2.1.2 [12] was used to call single-nucleotide polymorphisms. Outputs were used to generate consensus sequences.

**Phylogenetic Analyses**

Consensus sequences of the eight gene segments of the Nigerian virus were compared with the most related sequences available in GISAID (https://www.gisaid.org/) (Supplementary Table) and aligned by using MAFFT version 7 [13]. IQTREE version 1.6 was used to construct the maximum likelihood phylogenetic trees applying the best-fit general time-reversible model of nucleotide substitution with gamma-distributed rate variation among sites (GTR+F+I+G4) and performing ultrafast bootstrap resampling analysis (1000 replications) [14,15]. Phylogenetic trees were visualized by using FigTree version 1.4.2 (<http://tree.bio.ed.ac.uk/software/figtree/>).

**Intravenous Pathogenicity Index**

Ten six weeks-old Specific Pathogen Free White Leghorn chickens were inoculated following standard procedures [16]. Infected animals developed severe clinical signs and 100% mortality was observed within 72 hours after inoculation.

Animal experiment procedures were conducted in strict accordance with the Decree of the Ministry of Health n. 26 of 4 March 2014 on the protection of animals used for scientific purposes, implementing Directive 2010/63/EU, and approved by the Institute’s Ethics Committee (n. 5/2014).

**Reference**

1. Spackman E, Senne DA, Myers TJ *et al.* Development of a real-time reverse transcriptase PCR assay for type A influenza virus and the avian H5 and H7 hemagglutinin subtypes. J Clin Microbiol. 2002; 40: 3256–60.

2. Slomka MJ, Coward VJ, Banks J *et al.* Identification of Sensitive and Specific Avian Influenza Polymerase Chain Reaction Methods Through Blind Ring Trials Organized in the European Union. Avian Dis. 2007; 51: 227–234.

3. Slomka MJ, Pavlidis T, Banks J *et al.* Validated H5 Eurasian real-time reverse transcriptase-polymerase chain reaction and its application in H5N1 outbreaks in 2005-2006. Avian Dis. 2007; 51: 373–7.

**4**. Monne I, Ormelli S, Salviato A *et al.* Development and Validation of a One-Step Real-Time PCR Assay for Simultaneous Detection of Subtype H5, H7, and H9 Avian Influenza Viruses. J Clin Microbiol. 2008; 46: 1769–1773.

**5**. Hoffmann B, Hoffmann D, Henritzi D, Beer M, Harder TC. Riems influenza a typing array (RITA): An RT-qPCR-based low density array for subtyping avian and mammalian influenza a viruses. Sci Rep. 2016; 6: 27211.

**6**. Fusaro A, Zecchin B, Vrancken B *et al.* Disentangling the role of Africa in the global spread of H5 highly pathogenic avian influenza. Nat Commun. 2019; 10: 5310.

**7**. Zhou B, Donnelly ME, Scholes DT *et al.* Single-Reaction Genomic Amplification Accelerates Sequencing and Vaccine Production for Classical and Swine Origin Human Influenza A Viruses. J Virol. 2009; 83: 10309–10313.

**8**. Li H, Durbin R. Fast and accurate long-read alignment with Burrows-Wheeler transform. Bioinformatics. 2010; 26: 589–595.

**9**. Mckenna A, Hanna M, Banks E *et al.* The Genome Analysis Toolkit: A MapReduce framework for analyzing next-generation DNA sequencing data. Genome Res. 2010; 20: 1297–1303.

**10**. Depristo MA, Banks E, Poplin RE *et al.* A framework for variation discovery and genotyping using next-generation DNA sequencing data. Nat Genet. 2011; 43: 491–498.

**11**. Van Der Auwera GA, Carneiro MO, Hartl C *et al.* From FastQ data to high confidence variant calls: the Genome Analysis Toolkit best practices pipeline. Curr Protoc Bioinforma. 2014; **11**: 11.10.1–11.10.33.

**12**. Wilm A, Aw PPK, Bertrand D *et al.* LoFreq: a sequence-quality aware, ultra-sensitive variant caller for uncovering cell-population heterogeneity from high-throughput sequencing datasets. Nucleic Acids Res. 2012; 40: 11189–11201.

**13**. Katoh K, Standley DM. MAFFT Multiple Sequence Alignment Software Version 7: Improvements in Performance and Usability. Mol Biol Evol. 2013; 30: 772–780.

**14**. Thi Hoang D, Chernomor O, von Haeseler A, Quang Minh B, Sy Vinh L, Rosenberg MS. UFBoot2: Improving the Ultrafast Bootstrap Approximation. Mol Biol Evol. 2017; 35: 518–522.

**15**. Nguyen L-T, Schmidt HA, Von Haeseler A, Minh BQ. IQ-TREE: A Fast and Effective Stochastic Algorithm for Estimating Maximum-Likelihood Phylogenies. *Mol Biol Evol* 2014; **32**: 268–274.

**16**. OIE Terrestrial Manual, Chapter 2.1.14. Highly Pathogenic Avian Influenza. OIE Man Diagnostic Test Vaccines Terr Anim. 2004.

Supplementary Table. Similarities among the eight gene segments of A/duck/Nigeria/SK28T_19VIR8424-2/2019 and the 10 most related European H5N6 strains and the H5N8 strain from Nigeria (2016) obtained by the matrix of similarities resulted from the Maximum Composite Likelihood model in MEGA.

|  |  | A/duck/Nigeria/SK28T_19VIR8424-2/2019 | | | | | | | |
| --- | --- | --- | --- | --- | --- | --- | --- | --- | --- |
| GENE SEGMENTS | Accession numebers | HA | MP | NA | NP | NS | PA | PB1 | PB2 |
| A/Perigrine_falcon/Netherlands/18003274-001/2018_H5N6 | EPI_ISL_332439 | 99.1 | 98.9 | 98.8 | 98.8 | 98.9 | 98.7 | 99.2 | 98.8 |
| A/turkey/Germany-SH/AR185-L02549/2018_H5N6 | EPI_ISL_306989 | 99.1 | 99.0 | 98.7 | 98.8 | 98.4 | 98.7 | 99.2 | 98.6 |
| A/duck/Germany-SH/AR165-L02544/2018_H5N6 | EPI_ISL_305455 | 99.0 | 99.0 | 98.7 | 98.8 | 98.6 | 98.7 | - | 98.7 |
| A/chicken/Germany-SH/AR163-L02542/2018_H5N6 | EPI_ISL_305453 | 98.9 | 99.0 | 98.7 | 98.8 | 98.6 | 98.7 | 99.2 | 98.7 |
| A/white_stork/Germany-NI/AR251/2018_H5N6 | EPI_ISL_313226 | 98.9 | 98.9 | 98.8 | 99.0 | 98.7 | 98.7 | 99.1 | 98.8 |
| A/duck/Netherlands/17017236-001-005/2017_H5N6 | EPI_ISL_287906 | 99.1 | 99.1 | 98.8 | 99.1 | 98.5 | 98.9 | 99.2 | 98.9 |
| A/Eurasian_wigeon/Netherlands/1/2018_H5N6 | EPI_ISL_302824 | 99.1 | 99.1 | 98.8 | 99.2 | 99.0 | 98.9 | 99.2 | 98.9 |
| A/common_pochard/Germany-BY/AR09-18-L02421/2017_H5N6 | EPI_ISL_291109 | 99.1 | 99.1 | 98.8 | 99.2 | 98.9 | 98.8 | 99.3 | 98.9 |
| A/buzzard/Germany-NRW/AR279/2018_H5N6 | EPI_ISL_313227 | 99.1 | 99.1 | 98.8 | 99.0 | 98.7 | 98.8 | 99.2 | 98.7 |
| A/Tufted_duck/Netherlands/17017367-007/2017_H5N6 | EPI_ISL_288412 | 99.0 | 99.1 | 98.8 | 99.1 | 98.4 | 98.9 | 99.2 | 98.9 |
| A/duck/Nigeria/17RS737-43/2016_H5N8 | EPI_ISL_348279 | 98.1 | 97.4 | <50 | 94.2 | 97.1 | 93.5 | 97.9 | 90.9 |
